# Design of a Novel Electromagnetic Ultrasonic Transducer for Stress Detection

**DOI:** 10.3390/s25165205

**Published:** 2025-08-21

**Authors:** Changhong Chen, Chunguang Xu, Guangcan Yang, Yongjiang Ma, Shuangxu Yang

**Affiliations:** School of Mechanical Engineering, Beijing Institute of Technology, Beijing 100081, China; 3220225079@bit.edu.cn (C.C.); 3120225221@bit.edu.cn (G.Y.); 3120215244@bit.edu.cn (Y.M.); 3220235061@bit.edu.cn (S.Y.)

**Keywords:** residual stress, nondestructive testing, electromagnetic ultrasound, surface wave, sensor design

## Abstract

Accurate stress evaluation of structural components during manufacturing and operation is essential for ensuring the safety and reliability of advanced equipment in aerospace, defense, and other high-performance fields. However, existing electromagnetic ultrasonic stress detection methods are often limited by low signal amplitude and limited adaptability to complex environments, hindering their practical deployment for in situ testing. This study proposes a novel surface wave transducer structure for stress detection based on acoustoelastic theory combined with electromagnetic ultrasonic technology. It innovatively designs a surface wave transducer composed of multiple proportionally scaled dislocation meandering coils. This innovative configuration significantly enhances the Lorentz force distribution and coupling efficiency, which accurately measure the stress of components through acoustic time delays and present an experimental method for applying electromagnetic ultrasonic technology to in situ stress detection. Finite element simulations confirmed the optimized acoustic field characteristics, and experimental validation on 6061 aluminum alloy specimens demonstrated a 111.1% improvement in signal amplitude compared to conventional designs. Through multiple experiments and curve fitting, the average relative error of the measurement results is less than 4.53%, verifying the accuracy of the detection method. Further testing under random stress conditions validated the transducer’s feasibility for in situ testing in production and service environments. Owing to its enhanced signal strength, compact structure, and suitability for integration with automated inspection systems, the proposed transducer shows strong potential for in situ stress monitoring in demanding industrial environments.

## 1. Introduction

In the high-end equipment manufacturing industry, such as aerospace and weaponry, the lack of effective stress detection methods for components compromises product safety, reliability, and stability. Therefore, rapidly and accurately detecting component stress and understanding internal stress distribution, particularly in situ, is crucial for ensuring the structural safety, performance stability, and extended service life of high-end equipment [1,2,3,4]. Currently, there are various non-destructive testing techniques for stress detection, such as X-ray neutron diffraction methods, fiber Bragg grating methods, magnetic feature methods, ultrasonic methods, and resistance strain gauge methods. Although some of these methods offer various degrees of precision, when applied in harsh working environments in industrial settings during production or service, they still face numerous limitations, such as shallow detection depth, low sensitivity, or dependence on surface conditions and couplants [5,6,7,8]. To overcome challenges related to working environments, detection efficiency, and detection accuracy, electromagnetic ultrasonic stress detection technology has emerged as a promising method due to its advantages of non-contact, non-destructive, fast speed, and high sensitivity [9,10,11].

The core tool of electromagnetic ultrasonic testing is the electromagnetic acoustic transducer (EMAT), which generates Lorentz force within the component through electromagnetic induction, thereby exciting and receiving ultrasonic waves. The propagation speed of these waves changes according to variations in stress states, enabling electromagnetic ultrasonic testing for stress testing [12,13]. Since the 1960s, considerable progress has been made in EMAT development and application. Electromagnetic ultrasonic technology has been widely applied in the field of non-destructive testing [14]. Early work by Kawashima laid the groundwork for EMAT-based nondestructive evaluation [15,16], while subsequent designs by Wang and Murayama explored low-frequency excitation and selective mode generation [17,18]. More recent studies have focused on optimizing coil configurations, simulation methodologies, and signal analysis to enhance performance [19,20,21,22]. These include directionally focused transducers [20], creeping wave generation for cavity crack detection [22], and surface crack characterization using advanced finite element modeling [23]. In recent years, electromagnetic ultrasonic detection technology has gradually been applied in the field of stress detection. For example, experimental platforms developed by Muravyev, Zhang, and others have demonstrated the correlation between wave velocity and applied stress, validating the feasibility of EMATs for quantitative stress monitoring [24,25,26]. These studies indicate that the application of electromagnetic acoustic transducers in stress detection holds significant research value.

Despite this progress, traditional EMATs often suffer from limited energy conversion efficiency and low signal-to-noise ratios, which restrict their performance in industrial stress detection tasks. Therefore, optimizing electromagnetic ultrasonic transducers has become a focal point of current research, and enhancing signal strength and stability remains a key focus. Recent efforts have addressed this through innovations in coil design, such as optimizing turn geometry, implementing magnet arrays, and integrating flexible or electromagnet-based structures to improve performance under complex loading conditions [27,28,29,30]. Finite element analysis has become an essential tool in transducer design, enabling detailed investigation of Lorentz force behavior and acoustic field distribution [31,32].

Nevertheless, challenges persist in deploying EMATs for reliable stress evaluation under operational and production conditions. Current designs still exhibit limitations in sensitivity, directional control, and robustness to environmental variables [33]. To address these gaps, it is of great theoretical and practical value to systematically study the convenience, accuracy and reliability of EMAT in in situ detection environments. Based on this, this study aims to design and optimize a new type of efficient transducer, aiming at improving signal strength and measurement accuracy for stress detection. Through theoretical analysis, finite element simulation and experimental validation, the accuracy and reliability of the detection method will be evaluated in this study. The purpose of this study aims to provide an efficient, non-contact and field-deployable solution for stress detection in critical engineering applications, and to provide strong technical support and theoretical basis for the application of electromagnetic ultrasonic stress monitoring technology in practical engineering.

## 2. Basic Principles and Design of Electromagnetic Ultrasonic Transducer

### 2.1. Electromagnetic Ultrasonic Generation Model

**Figure 1:**
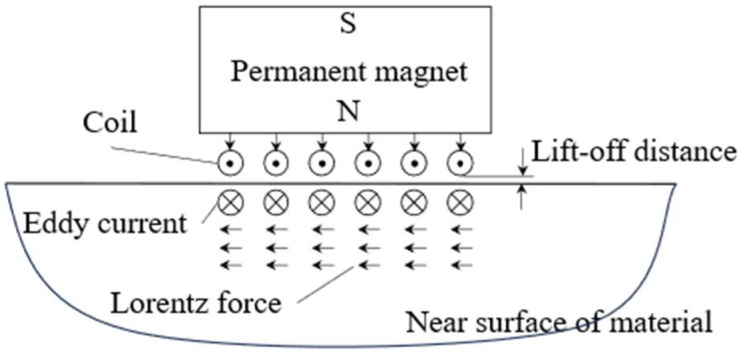
Schematic diagram of electromagnetic ultrasonic transducer.

EMATs generate and receive ultrasonic waves in materials through the Lorentz force. An alternating current in the excitation coil induces eddy current in the skin depth of the material. Under the action of static bias magnetic field, these currents will be affected by Lorentz forces, which will produce elastic disturbances and spread to material. Receive operates via the reverse process. Incident acoustic waves cause particle motion in the material, which under the bias magnetic field induces varying eddy current density. These currents interact with the coil, producing a measurable voltage in the receiving circuit. This reciprocity allows EMAT to serve in pulse-echo, pitch-catch, or through-transmission modes, similar to piezoelectric probes but without requiring physical coupling. A schematic of the electromagnetic ultrasonic generation mechanism is presented in Figure 1. The system is composed of multiple strands of copper wire positioned above a linear, homogeneous, and conductive material. And the permanent magnet within the transducer provides a static magnetic field perpendicular to the coil. When a high-frequency alternating current flows through the coil, a time-varying magnetic field varying with the current will be generated around the coil. According to Faraday’s law of electromagnetic induction, an induced current of opposite direction and the same frequency is generated on the surface of the material. Under the influence of the magnetic field, these induced currents interact with the magnetic field, producing Lorentz force. The Lorentz force, which drives elastic particle motion in the material, is given by:

(1) $$F_{L}=J_{e}\times B$$

where $F_{L}$ represents the magnitude of the Lorentz force generated by the magnetic field and induced current, $J_{e}$ is the induced current, and B is the magnetic flux density. The magnetic flux density B in the material is the sum of the static field $B_{c}$ generated by the permanent magnet and the dynamic field $B_{L}$ produced by the coil, which can be expressed as: $B=B_{c}+B_{L}$. In the case of relatively small currents in the EMAT, if the current is sufficiently small, $B_{L}$ can be neglected, and the magnetic flux density can be simplified to $B\approx B_{c}$ with only the larger static field directed towards the tested material.

The surface of the material serves as both the transducer area and can be viewed as the propagation region for ultrasonic waves, involving the multi-field coupling of electromagnetic fields and stress fields, with the Lorentz force being a key factor in this coupling. Under the influence of the Lorentz force, the material particles undergo elastic deformation, driving the surface particles to produce high-frequency vibrations, thus exciting ultrasonic waves on its surface, and the particle motion is described by:

(2) $$\nabla \cdot T+F_{L}=\rho \frac{\partial^{2}u}{\partial t^{2}}$$

where T represents the stress tensor, u is the displacement matrix, and ρ is the material density.

Equation (2) forms the core theoretical basis for the motion of material particles driven by the Lorentz force. This equation indicates that the behavior of the generated ultrasonic waves is dependent on the distribution of the induced current and the magnetic field.

Ultrasonic stress measurement mainly uses the acoustoelastic effect, which describes how the velocity of elastic waves varies with mechanical stress, which is usually represented by the acoustoelastic coefficient. For uniaxial loading and small stress levels, the relationship between wave velocity and stress can be linearized as:

(3) $$V_{\sigma }=V_{0}\left(1+A\sigma \right)$$

where $V_{0}$ represents the ultrasonic wave velocity in the absence of load, $V_{\sigma }$ is the velocity under the influence of load σ, and A is the acoustoelastic coefficient.

This relationship underpins the transducer’s ability to perform quantitative stress evaluation. This theoretical foundation is crucial for further optimizing the design of electromagnetic ultrasonic transducers and enhancing the accuracy of stress detection.

### 2.2. Surface Wave EMAT Design

**Figure 2:**
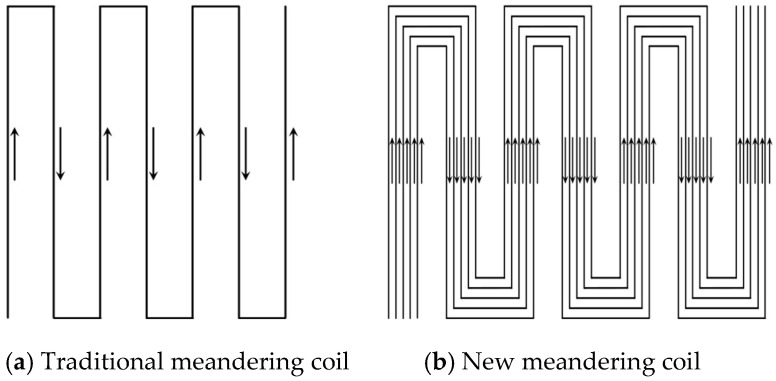
Schematic diagram of meandering coil. The direction of the arrow indicates the direction of current.

To efficiently obtain the ultrasonic surface waves, the EMAT coil structure must ensure optimal current distribution and Lorentz force orientation. The coil of conventional EMAT typically employs a meandering coil design, as shown in Figure 2a. This design ensures that adjacent conductors are spaced evenly and closely, allowing for uniform distribution of the induced current. The direction of the current in adjacent conductors is opposite, and thus the direction of the induced current is also opposite, resulting in opposing Lorentz forces. This design can effectively generate surface waves, enhance ultrasonic energy, improve the signal-to-noise ratio, and strengthen detection performance. When the material thickness exceeds four times the wavelength of the ultrasonic wave, it can be assumed that the high-frequency vibrations of the surface particles primarily form surface waves. In this case, the energy of the surface waves is concentrated at the material surface and subsurface, and it rapidly attenuates with increasing depth. In this study, the pitch-catch mode is employed, in which the transmitter and receiver are positioned at a fixed distance apart. This setup measures variations in the acoustic time-of-flight (TOF) between the two sensors, which are directly related to the applied stress via the acoustoelastic effect. The spatial separation between the transmitter and receiver helps suppress direct electromagnetic coupling noise, thereby enhancing the accuracy and reliability of stress evaluation. To achieve efficient surface wave excitation, the design typically requires that the spacing between adjacent conductors in the coil is half the wavelength of the ultrasonic wave, thereby effectively enhancing transducer efficiency and detection sensitivity.

In this study, a novel surface wave EMAT was developed by using a modified meandering coil structure shown in Figure 2b. This coil adds multiple proportionally reduced dislocation coils inside the traditional coil to optimize performance. This new configuration introduces multiple miniaturized coils, derived from proportional reduction of the original geometry and strategically offset along the scanning direction. This offset design can further enhance the strength and uniformity of the induced current distribution. The newly designed coil enhances the strength of the induced current and makes full use of the strong vertical magnetic field produced by the permanent magnet, thereby increasing the intensity of the vertical Lorentz force component and improving the excitation and reception efficiency of surface waves. Experiments show that the improved EMAT design with five parallel conductors significantly improves the Lorentz force and signal amplitude compared to the traditional design with a smaller number of conductors. Specifically, fewer than five wires result in lower Lorentz force density and weaker ultrasonic extrusion, whereas significantly more wires can increase coil inductance, reducing current amplitude for a given voltage and potentially causing heating and diminished efficiency. Beyond improving wave generation, these parameter optimizations also have a direct and positive impact on stress detection performance. Stronger and more uniform Lorentz-force excitation increases the amplitude and signal-to-noise ratio of received surface waves, making small stress-induced variations in propagation time easier to detect. This heightened sensitivity improves the accuracy of the acoustoelastic coefficient measurement, thereby enabling more reliable quantification of applied stress levels in metallic structures.

**Figure 3:**
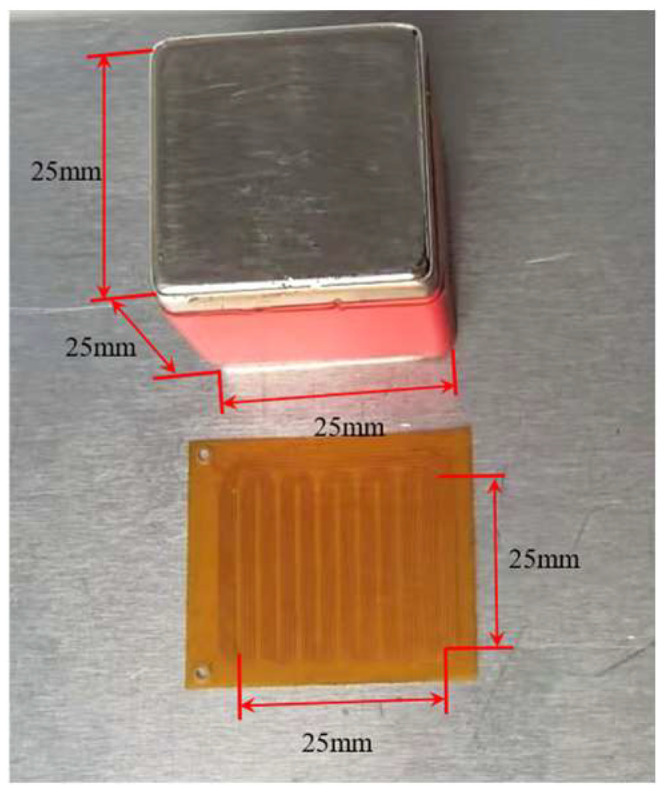
New electromagnetic ultrasonic surface wave transducer.

The final EMAT prototype shown in Figure 3 consists of a flexible printed circuit board (FPCB) coil and a magnet. The static magnetic field of this transducer is provided by a 25 mm × 25 mm × 25 mm cubic N52 neodymium-iron-boron (NdFeB) magnet. The FPCB coil consists of five turns of 0.08 mm diameter copper conductors. Each turn of wire is laterally offset by 0.08 mm. A pitch of 5 mm between coil periods was selected to match a design frequency of 0.6 MHz. The overall thickness is 0.2 mm, which ensures the coil’s lightweight, flexibility and minimal lift-off. The permanent magnet is placed directly on the coil structure, with the lift-off distance between the coil and the permanent magnet being 0.05 mm and 0.2 mm, respectively, forming a compact and rigid transducer assembly. To validate the design, two identical EMATs were fabricated and configured as a transmitter-receiver pair. The enhanced design aims to optimize surface wave excitation efficiency, signal strength, and detection stability under varying operational conditions.

## 3. Finite Element Simulation

### 3.1. Modeling

**Figure 4:**
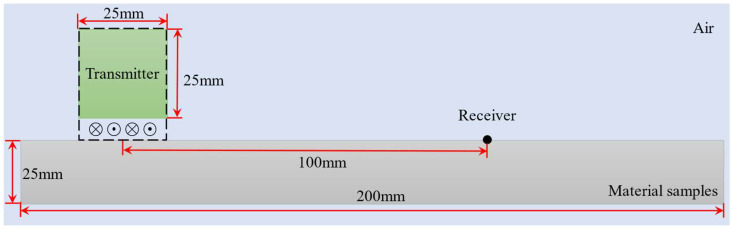
Schematic diagram of simulation modeling.

To investigate the performance of the proposed EMAT and its sensitivity to stress variation. This study employs the finite element analysis software COMSOL Multiphysics (Version 6.2) to establish a multiphysics simulation model for electromagnetic ultrasonic detection of plate specimens. The model aims to simulate the excitation, propagation, and reception of surface waves in a specimen plate under varying stress states. The design schematic of the simulation model is illustrated in Figure 4. The model geometry comprises four principal components: the test specimen, coil, permanent magnet, and a surrounding air domain. In the model, the specimen is a 6061 aluminum alloy plate with a length of 200 mm and a thickness of 25 mm. The static magnetic field is provided by a N52 NdFeB magnet with dimensions of 25 mm × 25 mm, while the excitation coil consists of five turns of copper wire with a diameter of 0.08 mm. The coil and magnet are both placed above the specimen, with the lift-off distances between the coil and the magnet being 0.05 mm and 0.2 mm, respectively, to simulate the lift-off gaps commonly found in real-world EMAT configurations. The specimen, permanent magnet, and coil are placed within a 400 mm × 150 mm air domain to simulate the sound wave propagation in a practical detection environment. The distance between the centers of the ultrasonic wave transmission and reception points is 100 mm, simulating the propagation process of the transmit and receive transducers within the plate specimen, enabling the measurement of TOF variations under different stress conditions.

The accuracy of material property data is critical during the simulation process. Table 1 lists the physical property parameters of the specimen material, including electrical conductivity, magnetic permeability, and elastic modulus.

The model enables detailed analysis of surface wave field distribution, propagation characteristics, and the investigation of the variations in surface wave propagation velocity under different stress conditions. This forms a basis for validating transducer performance and optimizing its structural parameters for enhanced stress sensitivity.

### 3.2. Simulation Process

The simulation was configured to reflect realistic excitation and measurement conditions. Table 2 lists the main parameters used during the simulation process, including the frequency of the electromagnetic ultrasonic transducer, the speed of sound in materials, the wavelength of the ultrasonic wave, and the amplitude of the excitation signal.

**Figure 5:**
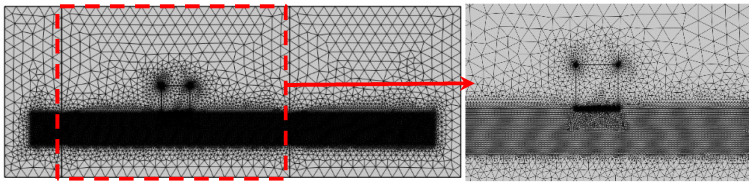
Grid division.

In the simulation model, the quality of the mesh elements directly affects the stability of the numerical solution. Therefore, during mesh division, it is essential to consider the type and size of mesh elements to ensure the numerical stability and computational accuracy of the model. A freely meshed triangular grid was employed throughout the simulation domain in Figure 5. The freely subdivided triangular mesh exhibits excellent adaptability, particularly suitable for simulations involving complex geometries and multiphysics coupling. In high-frequency simulations, smaller mesh sizes help improve result accuracy, but they also lead to an increase in computational load. Therefore, mesh division must strike a balance between accuracy and computational efficiency. In this simulation, based on the wavelength of the ultrasonic wave and the geometric characteristics of the specimen, at least 40 mesh elements are defined within the range of the ultrasonic wavelength, with a minimum mesh size of 0.12 mm, ensuring that the phase and amplitude variations of the sound wave during propagation can be accurately captured.

The choice of time step significantly affects the accuracy of simulation results and the stability of numerical solutions. This study employs the Generalized-α time-stepping method, which effectively improves the accuracy of time integration and possesses good numerical dissipation characteristics, making it suitable for solving dynamic responses in acoustic field problems. The setting of the time step must ensure that the propagation distance of the ultrasonic wave within one time step does not exceed the length of a single mesh element, and the size of the time step should follow the equation below:

(4) $$∆t\leq \frac{l_{min}}{C_{R}}$$

where ∆t represents the time step, $l_{min}$ is the size of the minimum mesh element, and c is the propagation speed of surface waves in the material.

This criterion ensures that the phase and amplitude variations of the sound wave can be adequately captured within each time step, thereby preserving waveform fidelity, avoiding numerical errors and instability in the computational results caused by excessively large time steps. In this simulation, for high-frequency acoustic field problems, due to the high sound speed and short wavelength, a time step of ∆*t* < 40 ns is selected to ensure computational accuracy. The simulation employs a 0.33 ns time step to ensuring numerical stability and accurate wave representation over the mesh elements, and to measure the small acoustic time changes caused by stress. Since 0.33 ns ≪ 40 ns, the simulation accurately captures signal dynamics without numerical errors.

The EMAT excitation was modeled by applying a sinusoidal burst signal to the coil, simulating the alternating current driving the transducer. The excitation function expressed as follows:

(5) $$f\left(t\right)=\left\{\begin{array}{c} \sin\left(2\pi f_{0}t\right),t\leq T_{0}\\ 0,T_{0}<t\leq 5T_{0} \end{array}\right.$$

where $f\left(t\right)$ is the applied load, $T_{0}$ is the period of the sinusoidal wave, and $f_{0}$ is the center frequency of the transducer.

The coil’s induced eddy current interacts with the static magnetic field to generate Lorentz forces, initiating ultrasonic wave propagation within the specimen.

### 3.3. Signal Analysis

**Figure 6:**
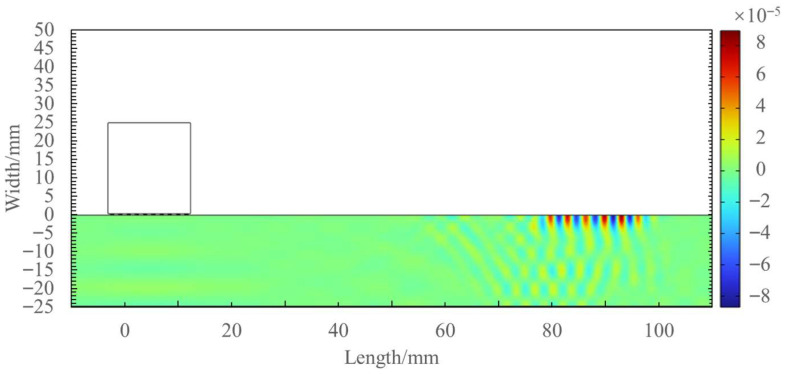
Simulation results.

After establishing the simulation model of the above novel electromagnetic ultrasonic transducer, the propagation process of ultrasound in the flat specimen under different stress states was simulated. The simulation results are shown in Figure 6, which illustrates the displacement generated by the propagation of ultrasound on the surface of the specimen. The simulated displacement field demonstrates that wave propagation is predominantly confined to the surface region, with a bright band observed within a depth of approximately λ and minimal energy transmitted through the thickness. Depth-resolved amplitude profiles confirm the rapid attenuation of wave energy below 1.5λ, consistent with the behavior of surface acoustic waves. Although bulk wave modes may be initially generated, they attenuate rapidly and contribute negligibly at the measurement depth. Ultrasonic surface waves are generated by the transmitting EMAT, travel along the plate surface, and are captured at the receiving point 100 mm away. When the stress state of the specimen changes, the wave speed also changes, which in turn affects the propagation time of the ultrasound over a fixed distance. By measuring the time difference in ultrasound propagation under different stress conditions, the stress variations in the specimen can be analyzed, thereby validating the effectiveness and sensitivity of the newly designed electromagnetic ultrasonic transducer in stress detection.

**Figure 7:**
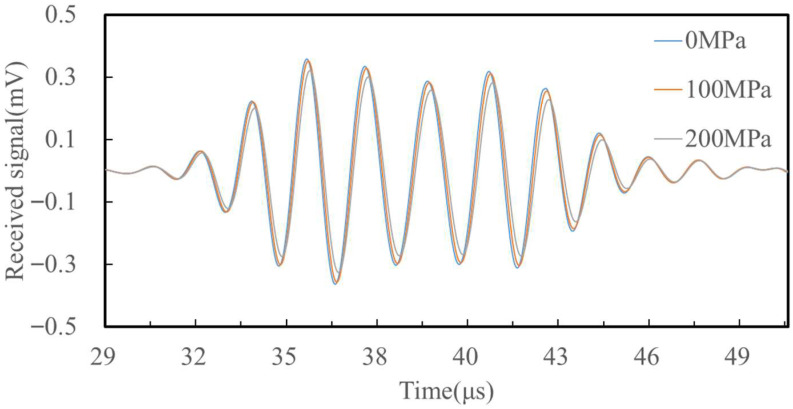
Simulation receiving signal. Waveforms for three stress states (0, 100, 200 MPa) are overlaid.

To analyze received waveforms, the point P (100, −0.1) mm was defined as the observation location. Figure 7 presents the received time-domain signals under three stress conditions: 0 MPa, 100 MPa, and 200 MPa. The first wave packet corresponds to the ultrasound under a stress state of 0 MPa, the second wave packet corresponds to the ultrasound under a stress state of 100 MPa, and the third wave packet corresponds to the ultrasound under a stress state of 200 MPa. As the applied stress increases, the arrival time decreases due to the acoustoelastic effect. The change in propagation time ∆t due to stress can be expressed as:

(6) $$∆t=t_{\sigma }-t_{0}$$

The corresponding stress change ∆σ is estimated using the linearized acoustoelastic relationship:

(7) $$∆\sigma =A\left(t_{\sigma }-t_{0}\right)$$

where $t_{\sigma }$ represents the propagation time under stress σ, and $t_{0}$ represents the propagation time in the absence of stress.

Simulation results show the following TOF values: *t*_0_ = 32.106 μs, *t*_100_ = 32.161 μs, *t*_200_ = 32.217 μs. Consequently, ∆*t*_100_ = 0.055 μs and ∆*t*_200_ = 0.111 μs. These small changes, though only fractions of a nanosecond, are accurately captured and validate the acoustoelastic model’s ability to detect stress-induced variations in surface-wave velocity. By quantifying the time shifts between the signals, the stress-dependent variation in wave velocity can be deduced, validating the stress detection capabilities of the proposed EMAT design.

## 4. Experimental Results and Discussion

### 4.1. Experimental System

**Figure 8:**
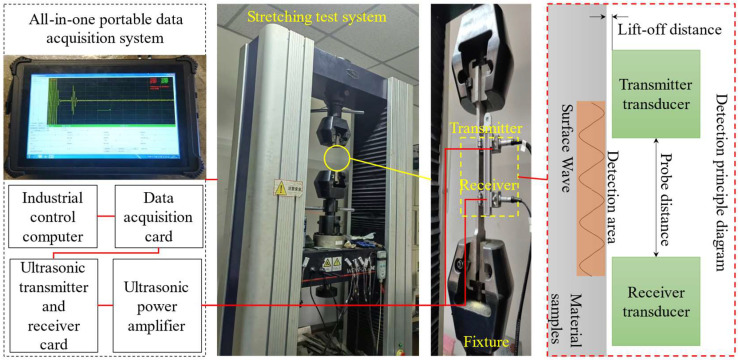
Experimental system.

To verify the performance of the newly designed electromagnetic ultrasonic transducer in stress detection, this study established an experimental system and conducted comprehensive experimental tests. The experimental apparatus and its connection arrangement are shown in Figure 8, and the system mainly consists of an integrated portable data acquisition system and a tensile testing system. The integrated portable data acquisition system includes the electromagnetic ultrasonic transducer, electromagnetic ultrasonic transmitter-receiver card, power amplifier, data acquisition system, and industrial computer. The electromagnetic ultrasonic transducer (Zhongke Innovation Technology Co., Ltd., Wuhan, China) is used to excite and receive ultrasonic waves. The electromagnetic ultrasonic transmitter-receiver card provides driving current to generate high-voltage pulse signals to excite the ultrasonic transducer. The power amplifier (Zhongke Innovation Technology Co., Ltd., Wuhan, China) is used to amplify signals with a gain range of 20 to 80 dB. The data acquisition system is used to record ultrasonic signals with a sampling rate of up to 200 MHz. The industrial computer controls the tensile testing machine and the electromagnetic ultrasonic transmitter-receiver card. The tensile testing system includes the tensile-compressive testing machine, specially designed fixtures, and standard tensile test specimens. The tensile-compressive testing machine (Sida Testing Technology Co., Ltd., Jinan, China) is used to apply different stress states to the test specimens. The fixtures use wedge-shaped blocks with a small angle, and the center of the fixtures coincides with the tensile axis to ensure that the tensile force is applied along the axis of the standard tensile specimen. The standard tensile specimens are made from the selected materials according to the corresponding standards.

In addition, since acoustic velocity and attenuation are sensitive to thermal variations, the entire experimental setup is placed in the laboratory, where the ambient temperature is stabilized within ±1 °C. A thermometer (Minchuang Electronics Co., Ltd., Shenzhen, China) with a resolution of 0.1 °C is used to control and monitor the measurement environment temperature. To avoid the impact of temperature variations on material attenuation and sound velocity, the temperature is maintained at a constant level of 24 (±1) °C during the stress measurement tests using central air conditioning.

The experimental steps and methods are as follows: First, select a 6061 aluminum plate with the same processing state as the corresponding model of the equipment, and prepare standard tensile specimens while marking the test points on the surface of the specimens to ensure consistent EMAT positioning. Next, use a specially designed fixture to securely mount the specimens on the tensile testing machine. Then, securely install the EMAT on the surface of the specimen, ensuring that it is accurately aligned with the marked test point to maintain consistent lift-off and coupling conditions. The transmitter and receiver EMATs were mounted on a rigid mechanical fixture that ensured a constant separation distance throughout the entire stress-loading cycle. Consequently, any observed variations in TOF can be attributed solely to stress-induced changes in wave velocity via the acoustoelastic effect, rather than to variations in propagation distance. Subsequently, use the tensile testing machine to incrementally load the tensile specimens, starting from an initial load of 0 MPa and gradually increasing to the yield strength limit stress value, while maintaining each load stable for 10 s after each increment of 30 MPa to achieve a uniform distribution of stress state. At each stable stress state, use the electromagnetic ultrasonic transmitter-receiver card to drive the EMAT to emit and receive ultrasonic signals, while recording the propagation time and amplitude of the ultrasonic waves through the power amplifier and data acquisition system. Finally, use the industrial computer to process the collected ultrasonic signals to extract the relationship curve between ultrasonic wave propagation speed and stress variation.

### 4.2. Test Results and Discussion

#### 4.2.1. Coil Transmission and Reception Experiment

**Figure 9:**
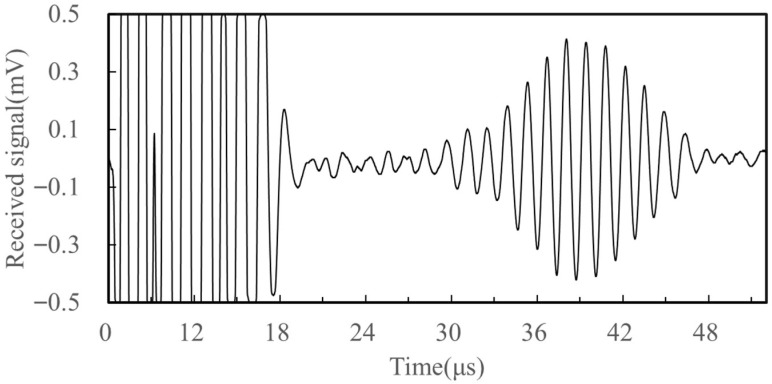
Received signal. Time-domain signals acquired under 0 MPa.

To assess the signal generation and reception performance of the newly developed EMAT, a surface wave propagation experiment was conducted with a transmitter–receiver spacing of 100 mm. Figure 9 presents a typical received waveform. The first wave packet is the crosstalk signal generated by the equipment, which is unavoidable; the second wave packet is the surface wave directly reaching the receiving transducer, and there are no significant other wave packets between the crosstalk signal and the direct wave packet. The absence of significant intermediate wave packets confirms that surface wave energy arrives without notable reflection or interference. Based on the distance between the transmitting and receiving transducers and the ultrasonic wave propagation time, the surface wave velocity can be calculated as 3125 m/s, with a relative error of 5.57% compared to the theoretical surface wave velocity of 2960 m/s in aluminum alloy. This indicates that the designed new electromagnetic ultrasonic surface wave transducer can effectively generate surface waves. Only surface waves are detected in the measurements, as both the EMAT structure and the receiving system are specifically optimized for the generation and reception of surface acoustic waves. While other wave modes may be excited in simulations, their energy rapidly attenuates during propagation and is effectively filtered out by the system.

**Figure 10:**
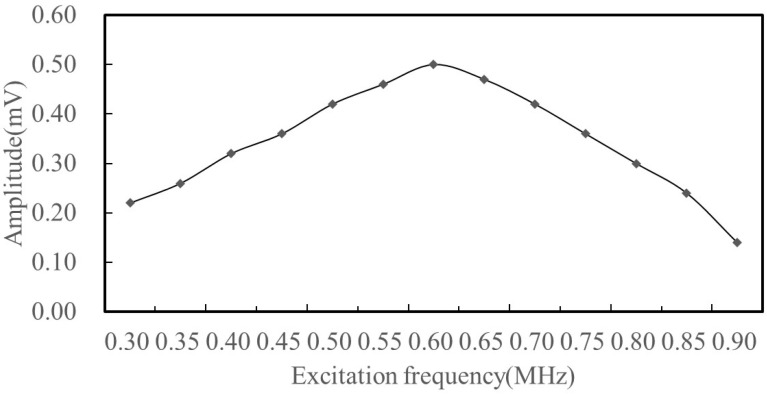
Frequency domain distribution.

It is well known that electromagnetic ultrasonic transducers can operate over a wide frequency range, and the amplitude of the generated signal reaches its maximum at the center frequency. The center frequency of the transducer designed in this study is 0.6 MHz. In order to verify the frequency response characteristics of the transducer, a frequency sweep test was conducted. The center frequency of the excitation signal is increased from 0.3 MHz to 0.9 MHz, with an interval of 0.05 MHz. The peak amplitudes at different emission frequencies were extracted from the received signals. Figure 10 shows the frequency response of the EMAT, the maximum amplitude appears near the excitation frequency of 0.6 MHz, which coincides with the designed center frequency. This indicates that the newly designed electromagnetic ultrasonic surface wave transducer in this study has good frequency response performance.

**Figure 11:**
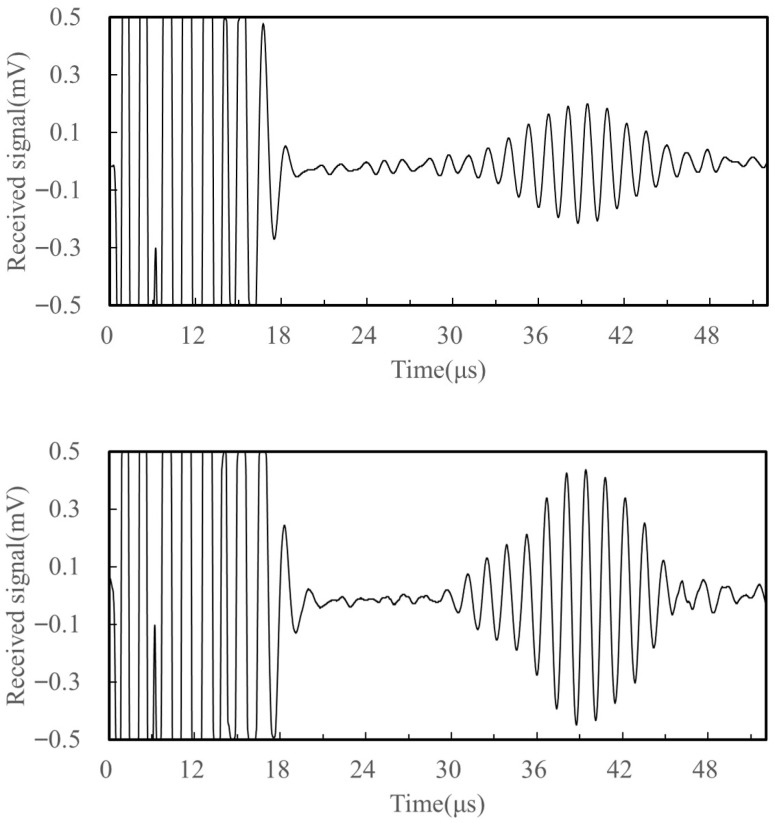
Comparison of received signals of two transducers.

At the same time, the traditional electromagnetic ultrasonic surface wave transducer was prepared for comparative testing. As shown in Figure 11, the received signal amplitudes of the traditional and new electromagnetic ultrasonic surface wave transducers were 0.18 mV and 0.38 mV, respectively. Experimental results indicate that changes in the coil directly affect the magnitude and distribution of the Lorenz force generated by the electromagnetic transducer. By introducing proportionally scaled-down dislocation coils, the new electromagnetic ultrasonic surface wave transducer significantly enhanced the electromagnetic coupling efficiency, resulting in a 111.1% increase in the received signal strength. Adjusting the coil parameters of the new transducer improves the efficiency of surface wave generation, thereby increasing the acoustic-bomb sensitivity and making the flight time changes caused by stress more obvious. This optimized design makes full use of the strong magnetic field at specific locations, improving the quality and stability of signal reception and laying a foundation for further enhancing the reliability of electromagnetic ultrasonic detection technology.

#### 4.2.2. Stress Detection Experiment

**Figure 12:**
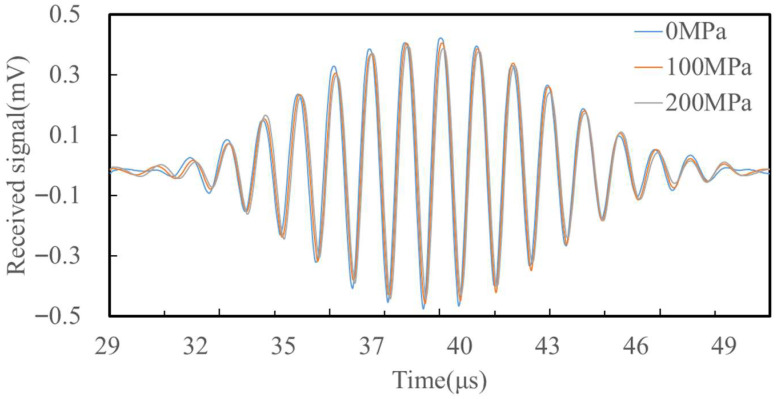
Received signals under different stress conditions. Received signals under 0, 100, and 200 MPa stress.

**Figure 13:**
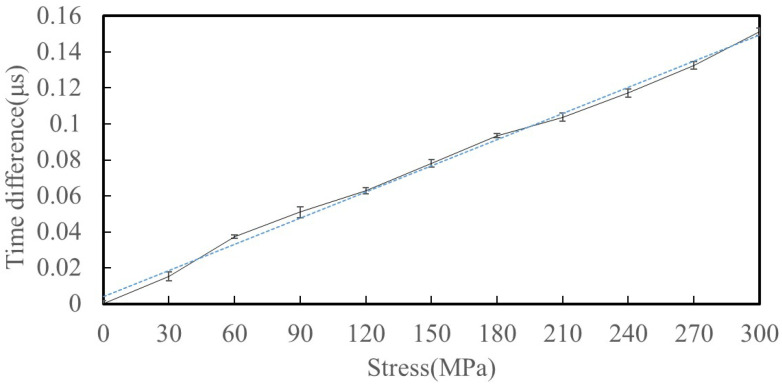
Linear relationship between time difference and stress. Each point denotes ∆*t* value at 30 MPa intervals. The dashed line denotes the linear regression fit.

Based on the experimental steps in Section 4.1, tensile specimen testing was conducted, and Figure 12 shows the received waveforms at loads of 0 MPa, 100 MPa, and 200 MPa. The data indicate that the ultrasonic wave propagation speed varies significantly with the increase in applied stress. Figure 13 presents the experimental results at 30 MPa increments from 0 MPa to 300 MPa, and plots the extracted wave propagation time difference as a function of applied axial stress. It can be observed that as the stress increases, the ultrasonic wave propagation time shows a linear increasing trend, and there is a strong correlation between the surface wave velocity and axial stress, which is consistent with theoretical expectations. This phenomenon can be explained by the effect of stress on the material’s elastic constant: an increase in stress leads to changes in the internal microstructure of the material, which in turn affects the propagation speed of ultrasonic waves. Specifically, when the stress increases from 0 MPa to 300 MPa, the ultrasonic wave propagation time increases by approximately 0.152 μs. These findings confirm the sensitivity of ultrasonic surface waves to internal stress states in metallic materials. Additionally, the small standard deviation of the experimental data indicates that the measurements have high repeatability and reliability.

#### 4.2.3. Stress Prediction Experiment

The calculated slope of the linear fitting in Figure 13 is 68.493, which serves as the acoustoelastic coefficient of the 6061 aluminum plate and serves as the basis for the stress prediction model. This coefficient was used to calibrate the EMAT for practical applications. A separate test was conducted using a specimen subjected to randomly selected tensile loads. During the testing period, each point is maintained stable for 10 s for detection purposes. Table 3 summarizes the comparison between the predicted stress values obtained using the proposed EMAT and the actual stress applied via the tensile testing machine. A total of 30 groups of axial stress were measured. For comparison, a traditional EMAT was also tested under identical conditions. The traditional transducer exhibited significantly lower signal amplitude and less consistent wave propagation time, leading to increased measurement uncertainty and larger deviations in predicted stress.

It can be seen from 30 test cases that the stress state of the specimen can be predicted, and the stress value can be determined based on the surface wave propagation time. As shown in Table 3, the relative error between the detected and actual stress values ranges from 0.95% to 10.36%, the average error is 4.53%, the average absolute error is 6.16 MPa, and the average median error is 5.1 MPa, which is a significant improvement compared with the traditional electromagnetic ultrasonic method with an average error of more than 10.07%, an average absolute error of 11.59 MPa, and an average median error of 8.6 MPa. This performance enhancement is primarily attributed to the improved coil design, which increases electromagnetic coupling efficiency and reduces signal noise. In this experiment, the main sources of error include calibration errors of the sensor, variations in environmental temperature, and data random fluctuations caused by electronic and environmental noise. Nevertheless, compared to conventional transducers, the novel EMAT demonstrates superior accuracy, measurement stability, and practical feasibility for nondestructive residual or applied stress assessment in aluminum alloy structures.

### 4.3. Validation on 6061 Aluminum Alloy Specimens

To further validate the practical applicability of the proposed electromagnetic ultrasonic stress detection method, an experiment was conducted on a 6061 aluminum alloy panel (Taiming Aluminum Manufacturing Co., Ltd., Wuxi, China) used as a pre-machined blank for aircraft components. The test specimen was a flat thin-walled panel, representative of critical structural parts in aerospace applications, where residual stress assessment is essential for fatigue and safety monitoring.

**Figure 14:**
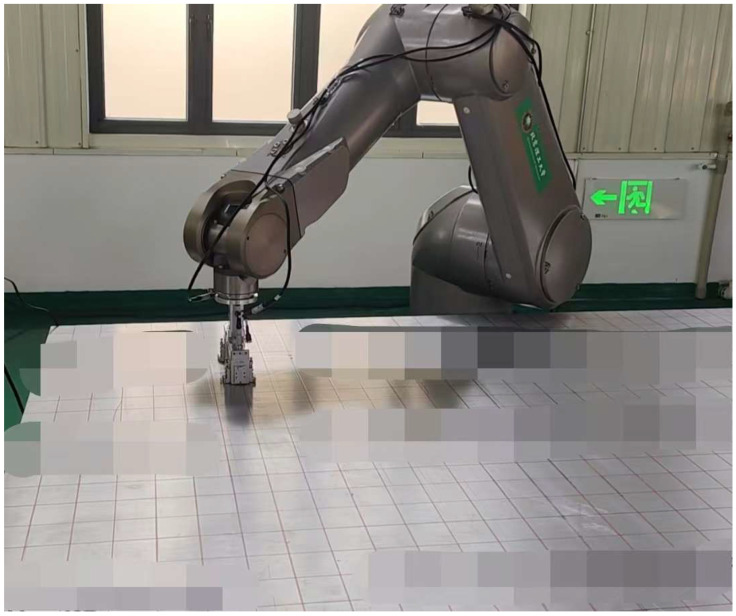
6061 aluminum alloy sample verification photos.

As shown in Figure 14, the inspection was performed in an automated setup using a robot system equipped with a high-precision linear positioning stage. The EMAT developed in this study was integrated into the robot end-effector to perform automated scanning of the specimen surface. The scanning covered the full area of the panel, and measurements were taken at intervals of 50 mm to construct a spatially resolved stress distribution map.

**Figure 15:**
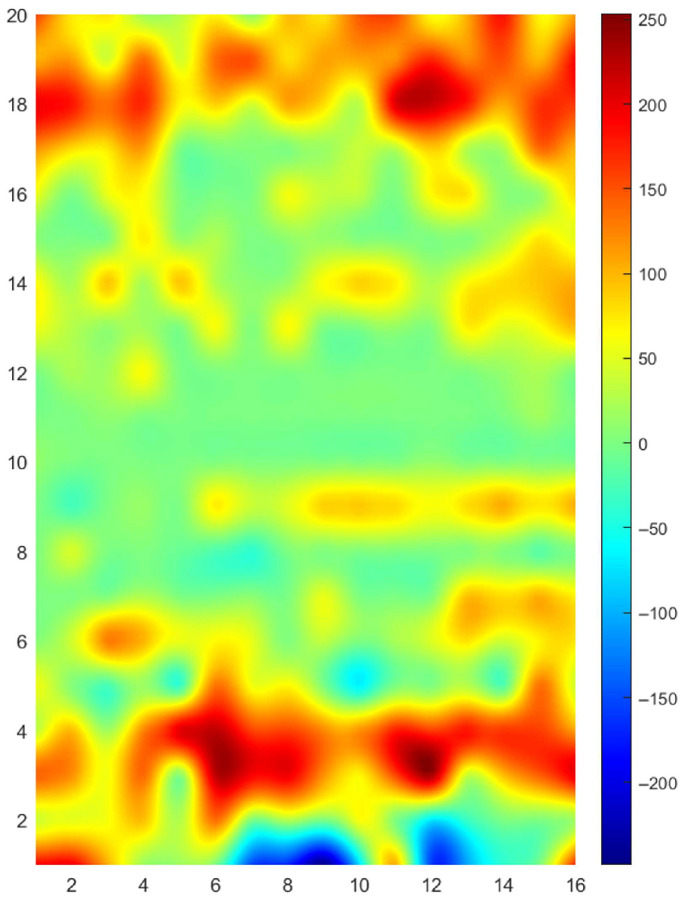
Stress distribution contour map. Stress distribution based on ∆*t* data.

During the test, the surface wave velocity was measured at each scanning point. Based on the previously established acoustoelastic model, these velocities were converted into local stress values. The resulting data were compiled to generate a stress distribution contour map, as shown in Figure 15. The results revealed clear stress concentration regions near the edge boundaries zones, consistent with expectations for residual stress patterns from forming. Future work will focus on miniaturizing the EMAT geometry to enable localized measurements (30 mm), thereby improving spatial resolution without affecting measurement validity.

To ensure the accuracy and reliability of the results, cross-validation was performed using two independent stress measurement techniques: the hole-drilling method and X-ray diffraction (XRD). The spatial distribution and magnitude of the detected stresses using electromagnetic ultrasonic testing showed strong agreement with those obtained from the hole-drilling method and XRD, with average deviations within 7.2%. A comparative summary of the results is provided in Table 4.

As shown in Table 4, the stress values obtained by the three methods exhibit good numerical agreement across different measurement points. The average relative deviation between the EMAT-based results and those obtained by the hole-drilling method is 2.39%, while the deviation from XRD results is 1.26%. For instance, at measurement point P1, the EMAT-detected stress was 142.3 MPa, compared to 146.0 MPa measured by the hole-drilling method, corresponding to a deviation of 3.7 MPa or 2.53%. The stress measured by XRD at the same point was 143.5 MPa, yielding a deviation of only 1.2 MPa or 0.84% from the EMAT result. Similar consistency was observed at other points, indicating that the EMAT technique provides high accuracy and measurement stability in multi-point stress distribution detection.

These results confirm the capability of the proposed method for non-contact, full-field, in situ stress evaluation in complex, real-world aerospace components. The integration with robotic platforms further demonstrates its potential for intelligent, automated inspection in manufacturing and maintenance workflows.

## 5. Conclusions

This study designed a novel surface wave EMAT and stress detection method based on proportionally scaled dislocation coils. Finite element simulation was used to study the acoustic field characteristics and simulate the propagation behavior of ultrasonic waves during the detection process. Experimental verification proves the superior performance of the new EMAT. The proposed coil design significantly enhances the Lorentz force efficiency, thereby improving the ultrasonic excitation performance, as well as extracting the received waveform to precisely detect surface stress in the specimen. Compared to traditional surface wave EMAT, the newly designed system shows a significant increase in received signal strength, with a 111.1% improvement, exhibiting a higher signal-to-noise ratio and stability. In the stress measurement experiment, the ultrasonic wave speed was first measured under stress-free conditions, and then a stress prediction verification experiment was performed. The experimental results indicate that this EMAT can accurately detect stress within the material, with an average detection error of 4.53%. Furthermore, the system was applied to the inspection of a 6061 aluminum alloy wall panel used as a blank in aircraft structural components. The stress distribution obtained by the EMAT method showed high spatial resolution and strong consistency with reference data obtained from the hole-drilling method and XRD. Fully validating its reliability in the field of stress detection. Compared with traditional ultrasonic methods, this system has significant advantages in measuring surface stress of components without the need for coupling agents, and is particularly superior to traditional electromagnetic ultrasonic testing methods in terms of signal strength improvement and detection stability. The innovative design of this study not only enhances the practicality of electromagnetic ultrasonic technology but also lays the foundation for the promotion and application of non-destructive testing technology in stress detection in industrial sites. This achievement provides a more efficient and reliable technical means for solving stress problems in engineering structures, further demonstrating the application potential of the novel surface wave EMAT as a non-destructive testing tool.

## Figures and Tables

**Table 1:** Material property parameters.

Property	Name	Value	Unit
Electrical conductivity	sigma	$2.6\;\times \;10^{7}$	S/m
Magnetic permeability	mur	1.0003	_
Relative permittivity	epsilonr	1.0	_
Young’s modulus	E	68.9	GPa
Density	rho	2.70	$g/cm^{3}$
Poisson’s ratio	nu	0.33	_

**Table 2:** Simulation parameters.

Property	Name	Value	Unit
Transducer frequency	$f_{0}$	0.6	MHz
Longitudinal wave velocity in aluminum	CL	6299	m/s
Short wave velocity in aluminum	CS	3150	m/s
Surface wave velocity in aluminum	SH	2960	m/s
Wavelength in aluminum	λ	4.93	mm
Number of grid cells per wavelength	N	41	_
Step size	Δt	0.33	ns
Grid size	l	0.12	mm

**Table 3:** Stress prediction data.

Point	Actual Value	New Transducer Detected Value	New Transducer Absolute Error	New Transducer Relative Error	Traditional Transducer Detected Value	Traditional Transducer Absolute Error	Traditional Transducer Relative Error
1	110.9	118	7.1	6.40%	113	2.1	1.89%
2	200.0	191	9.0	4.50%	177	23	11.50%
3	190.0	184	6.0	3.16%	196	6.0	3.16%
4	135.6	139	3.4	2.51%	129	6.6	4.87%
5	250.4	241	9.4	3.75%	260	9.6	3.83%
6	50.1	46	4.1	8.18%	34	16.1	32.14%
7	35.7	32	3.7	10.36%	51	15.3	42.86%
8	115.9	111	4.9	4.23%	106	9.9	8.54%
9	125.4	128	2.6	2.07%	113	12.4	9.89%
10	45.5	50	4.5	9.89%	53	7.5	16.48%
11	140.5	154	13.5	9.61%	168	27.5	19.57%
12	300.2	304	3.8	1.27%	316	15.8	5.26%
13	195.1	205	9.9	5.07%	200	4.9	2.51%
14	290.3	295	4.7	1.62%	289	1.3	0.45%
15	215.4	212	3.4	1.58%	225	9.6	4.46%
16	160.4	148	12.4	7.73%	139	21.4	13.34%
17	245.3	240	5.3	2.16%	238	7.3	2.98%
18	170.8	161	9.8	5.74%	150	20.8	12.18%
19	210.9	203	7.9	3.75%	209	1.9	0.90%
20	100.1	106	5.9	5.89%	116	15.9	15.88%
21	295.8	293	2.8	0.95%	296	0.2	0.07%
22	205.6	202	3.6	1.75%	215	9.4	4.57%
23	305.1	301	4.1	1.34%	319	13.9	4.56%
24	180.1	189	8.9	4.94%	191	10.9	6.05%
25	130.3	135	4.7	3.61%	126	4.3	3.30%
26	240.3	232	8.3	3.45%	249	8.7	3.62%
27	220.8	217	3.8	1.72%	234	13.2	5.98%
28	120.0	113	7.0	5.83%	106	14.0	11.67%
29	70.4	66	4.4	6.25%	47	23.4	33.24%
30	90.9	85	5.9	6.49%	76	14.9	16.39%

**Table 4:** Comparison of stress test results (unit: MPa).

Points	EMAT	XRD	Hole-Drilling	Relative Error (XRD)	Relative Error (Hole-Drilling)
P1	142.3	143.5	146.0	0.84%	2.53%
P2	165.1	164.0	161.8	0.67%	2.04%
P3	121.7	123.0	125.0	1.06%	2.64%
P4	187.6	189.3	192.0	0.90%	2.29%
P5	133.4	131.0	130.2	1.83%	2.46%
Average	_	_	_	1.26%	2.39%

## Data Availability

All data supporting the findings of this study are available within the paper.
